# Diversity strengthens competing teams

**DOI:** 10.1098/rsos.211916

**Published:** 2022-08-10

**Authors:** J. Rowlett, C. J. Karlsson, M. Nursultanov

**Affiliations:** ^1^ Mathematical Sciences, Chalmers University of Technology and the University of Gothenburg, 41296 Gothenburg, Sweden; ^2^ Department of Mathematics and Statistics, University of Helsinki, PO Box 68, Helsinki FI-00014, Finland

**Keywords:** equilibrium strategy, game theory, diversity, competition, biodiversity

## Abstract

How does the composition of a collection of individuals affect its outcome in competition with other collections of individuals? Assuming that individuals can be different, we develop a model to interpolate between individual-level interactions and collective-level consequences. Rooted in theoretical mathematics, the model is not constrained to any specific context. Potential applications include research, education, sports, politics, ecology, agriculture, algorithms and finance. Our first main contribution is a game theoretic model that interpolates between the internal composition of an ensemble of individuals and the repercussions for the ensemble as a whole in competition with others. The second main contribution is the rigorous identification of all equilibrium points and strategies. These equilibria suggest a mechanistic underpinning for biological and physical systems to tend towards increasing diversity due to the strength it imparts to the system in competition with others.

## 1. Introduction

Diversity is a ubiquitous concept of great importance in multiple fields including scientific research [1], education [2–4], human resource management [5,6], business [7,8], sports [9], politics [10], ecology [11–17], algorithms [18], networks [19,20], finance [21–29] and agriculture [30–33]. In each of these contexts, diversity may take on a different meaning. Here, we broadly use *diversity* as a flexible concept for *anything* that *differentiates*.

For groups of people, diversity includes not only demographic differences [1] but also deep diversity like personality, mentality and past experiences [34]. One of the reasons diversity may be beneficial in research is that teams with members from diverse backgrounds may have a greater variety of perspectives [1]. A larger research group may be more likely to present a correct analysis and to draw reliable conclusions if all group members contribute to a rigorous internal review process. Quoting [35] ‘There is growing evidence that embracing diversity—in all its senses—is key to doing good science’. In [36], a quantitative study of over 9 million papers and 6 million scientists, those authors found ‘that ethnic diversity resulted in an impact gain of 10.63% for papers, and 47.67% for scientists’. According to [1], ‘a paper generated by a more diverse research group could tap into different networks and thus attract greater attention and citations, an effect observed in patents studies [37], and in inter-institution and international collaborations [38]’.

For similar reasons diversity may be broadly beneficial in education as explored in [2–4]. Quoting [39] ‘researchers have documented that students’ exposure to other students who are different from themselves and the novel ideas and challenges that such exposure brings leads to improved cognitive skills, including critical thinking and problem solving’. In human resources [5,6] and business [7,8], the effects of diversity upon performance have also been investigated. A study of 385 Norwegian companies [34] analysed the benefits of deep level diversity in a corporate context. This study provided strong support for the notion that the higher the level of board diversity with respect to the board members’ backgrounds (both professional and personal) and personalities, the higher the degree of board creativity and cognitive conflict during the decision-making process. They proposed that the deep level diversity of members may result in a board that possesses a greater set of skills, competencies and perspectives. For similar reasons, diversity may be beneficial in political contexts [10]. Similarly, in the context of professional sports, meta-analyses showed that overall group diversity has a positive effect on group outcomes [9,40]. A diverse sports team with a broad skill set may be able to outcompete a team with a narrow skill set by exploiting those skills which are lacking in a team with less diversity across its members.

A group of people with a diverse skill set can manage a wider array of challenges and thereby benefit from diversity. In some cases, diversity is even more crucial; there are biological examples in which diversity is required for survival. For example, in a biofilm different organisms coexist in a symbiotic form, because the different species produce chemicals that other species require but do not necessarily produce themselves [41]. Another example of the importance of biodiversity in an ecological system is provided by the human microbiome. The gut bacterial ecosystem is important for health, not only digestive and metabolic function, but also cardiovascular and neuropsychiatric health [42]. Public databases estimate on the order of 10 000 bacterial species in this ecosystem [43]. Reduced gut biodiversity is associated with health impairment such as Crohn’s disease [44].

More generally, the overall health of an ecological system is often judged by its level of biodiversity [42]. This biodiversity can be measured at different levels ranging from an ecosystem comprising different species [42] to a single species comprising phenotypically variable individuals [45]. One instance of tremendous biodiversity is provided by marine microbes. Their species diversity is estimated to exceed 200 000 species in the plankton [46,47]. At all levels of taxonomy, from species to intra-strain comparisons, there exists a tremendous variability in genetic, physiological, behavioural and morphological characteristics [48–62]. In [16,45,63], we suggested that this phenotypic heterogeneity in all microbe organisms is what makes it possible for countless microbe species to coexist and for new species to continually emerge [64]. Inspired by the natural evolution of species, evolutionary algorithms use operations like mutation, recombination and selection to evolve a multi-set of solutions over time. Population diversity is crucial to these algorithms, perhaps similar to its importance in biological systems [18]. In some sense, just as the diversity of a research team may contribute to their ability to create and innovate, biodiversity may play a similar role for ecological systems by facilitating ecological innovation. The richer the diversity of life, the greater the opportunity for medical discoveries, economic development and adaptive responses to new challenges [30–33].

On the one hand, we may view diverse individuals as having different strengths, but on the other hand, we could also view diverse individuals as having different weaknesses. From the perspective of diverse individuals having different weaknesses, diversification can be a method to mitigate the risk associated with too many individuals having the same weakness. This is, for example, beneficial to designing power grids and networks [19,20]. Presumably, every investor is also familiar with the concept of risk mitigation through diversification of investment products. In finance, a cornerstone of modern investment strategies, developed by Harry Markowitz in the 1950s [65,66], is known as *modern portfolio theory*, for which Markowitz received the Nobel Prize in Economics in 1990. The prize recognized his development of a rigorous operational theory for portfolio selection under uncertainty which has evolved into a foundation for financial economics research. A key concept in modern portfolio theory is to simultaneously analyse two dimensions: the expected return on the portfolio and its variance. Based on Markowitz’s work, an investor can construct a portfolio of multiple assets to maximize returns for a given level of risk. Conversely, given a desired level of expected returns, the investor can construct a portfolio with the lowest possible risk. Although it may seem unrelated, a similar approach towards risk mitigation has been suggested in agriculture [30–33]. In these works, they observe that crop diversification, similar to portfolio diversification, may become increasingly important the context of climate change.

In all of these contexts, diversity is beneficial for specific reasons. Some of these reasons are heuristically similar, but we are not aware of one clear mechanistic underpinning for all of them. As a step in this direction, to investigate the strength of diversity in a broad sense that can be applied to many contexts, we turn to theoretical mathematics. The advantage is that theoretical mathematics is not constrained to any one specific application. The limitation is that simplifying assumptions must be made to obtain results, and so a theoretical mathematical model will never be a perfect real-world match. However, the same holds for all fundamental science, and one cannot deny its utility.

Here, we study collections of individual entities which compose a team. Then, we investigate competition between such teams. All teams must obey a set of rules. Game theory sets a natural mathematical foundation to analyse such situations. Harnessing the tools of game theory requires a mechanism for interpolating from interactions between individuals to team-level repercussions. To investigate teams comprising unique and possibly diverse individuals, we introduce a mathematical model that quantifies how the composition of the individuals within a team affects its competition with other teams. We then identify the Nash equilibrium points and strategies in the game theoretic model. These strategies are characterized by diverse teams, suggesting a mechanistic underpinning for the strength of diversity due to the competitive advantage this diversity imparts to the team.

## 2. Results

The games of teams we introduce here generalize the game theoretic competitive model Rowlett *et al.* introduced in [16,45,63]. In [45,63], a major aim was to interpolate from individual competitions between microbes to the cumulative consequences for the species. However, there is no mathematical reason that the individual competitors in that model must be microbes, or anything else for that matter. One of the strengths of theoretical mathematics is that it is not constrained to specific applications. Consequently, a model developed with one application in mind may prove useful for numerous other contexts. The game theoretic model we construct here is a significant generalization of the model developed in [45,63]. We consider the model itself to be a meaningful contribution and therefore in itself a result because it is a tool that can be applied to any collection of individuals that compete with other collections of individuals, whether they are people, animals, microbes, investment products, or anything else. Our model could be combined with other competition models for teams to enhance them by assessing the team-level consequences of incorporating diversity among the individuals composing the teams.

### 2.1. Games of teams

We offer a heuristic explanation of our games of teams before providing the rigorous mathematical definitions. A collection of teams comprising individuals compete. One can imagine this as an event between the teams in which each team puts forth one player whose competitive ability is determined according to the team’s strategy. A constraint on all teams is imposed which limits the mean competitive ability assessed over all players of the team. This can correspond to a budget constraint, or a resource constraint, or reflect the fact that individuals cannot always perform their best and can make mistakes. The player is paired with a randomly selected opponent from a randomly selected competing team. The opponent’s competitive ability is determined according to their team’s strategy. The competitor with the higher competitive ability wins this round of play. The competition continues, and cumulative wins and losses are assessed and used to define the payoffs to all of the competing teams.

To reduce this situation to mathematical expressions and analyse them, we begin by identifying a specific *competitive ability* with a real value x∈R. Simply put, *x* beats anything lower and is beaten by anything higher; the same value is a tie. The competitive ability is a versatile concept that can be adapted to each specific field of application. It could be used to quantify one specific characteristic that is pertinent to competition, or it could be used to represent an aggregate assessment across all competition-relevant characteristics. The competitive ability could also be used to represent resource allocation within a team. A *strategy* is a rule for assigning competitive abilities to the individuals that compose the team subject to a constraint that may correspond to biological or financial limitations. We may at times abuse notation by identifying a team with its strategy.

**Definition 2.1 (Bounded measurable and continuous strategies).**

Let *f* be a non-negative, bounded, Lebesgue measurable function that is not identically zero and is compactly supported. Then, such a function *f* is known as an $L^{\infty }$ strategy, or equivalently, as a bounded measurable strategy. If we further assume that *f* must be continuous, then it is known as a continuous strategy. For a strategy *f*, we define

2.1 $$F(x)=\int_{-\infty }^{x}f\;(t)\;dt=\int_{\left[-\infty ,x\right]}f\;d\mu ,\;\|f{\|}_{L^{1}}=\int_{-\infty }^{\infty }f\;d\mu ,$$

with integration respect to the Lebesgue measure, *μ*. All strategies will be assumed to satisfy the constraint on the mean competitive ability, abbreviated MCA, which is defined as

2.2 $$\mathrm{MCA}(f)=\frac{1}{\|f{\|}_{L^{1}}}\int_{-\infty }^{\infty }tf\;(t)\;dt\leq C,\;\mathrm{for}\;a\;\mathrm{fixed}\;C\in R.$$

The corresponding competitive games are known as the *bounded measurable game of teams* and the *continuous game of teams*, respectively. We will also analyse *discrete strategies* and a corresponding *discrete game of teams*.

**Definition 2.2 (Discrete strategies).**

Let *M* > 0 be fixed. A discrete strategy is a non-negative function on the discrete set of competitive abilities${\left\{x_{j}=\frac{j}{M}\right\}}_{\;j\in Z}$

$$A\;:\;{\left\{x_{j}\right\}}_{\;j\in Z}\to [0,\infty ).$$

We assume that *A* has finite support, and $|A|=\sum_{\;j\in Z}A(j/M)>0$. All strategies will be assumed to satisfy the constraint on the mean competitive ability

$$\mathrm{MCA}(A)=\frac{1}{\left|A\right|}\sum_{\;j\in Z}A\left(\frac{\;j}{M}\right)\frac{j}{M}\leq C,\;\mathrm{for}\;a\;\mathrm{fixed}\;C\in R.$$

The game in this case is the *discrete game of teams*. In the discrete game, competitive abilities may only be integer multiples of 1/*M*, so in this way one can view 1/*M* as a single unit of competitive ability. The strategy is a rule for assigning the competitive abilities of the team members, but we note that this does not mean that each team member’s competitive ability is constant over time. The competitive abilities of the individuals can vary while maintaining a given strategy for the team as a whole. If one normalizes the strategy in the bounded measurable and continuous games by dividing by the *L*^1^ norm, then the strategy can be understood as a probability density function. Similarly, in the discrete game, dividing by |*A*|, the strategy can be interpreted so that the value at each discrete competitive ability is the probability that a randomly selected individual is assigned that competitive ability. From this perspective, the *mean competitive ability* is the first moment of the probability density function (strategy). It is necessary to impose a constraint on the MCA because otherwise one would simply seek strategies supported as close to ∞ as possible. This would correspond to unlimited resources or infallible super-individuals and is not realistic. We suggest that it is reasonable to assume that strategies are compactly supported, because in all practical applications of which we are aware, this will always be the case. Subject to a constraint on the mean competitive ability, what is the best way to assign competitive abilities to the individuals of a team? Equivalently, what is the best way to allocate resources to the members of a team, subject to a constraint on the total amount of resources available? To quantify the success of different strategies in competition, we define their game theoretic payoffs. We will then use these payoff functions to search for strategies that cannot be defeated.

#### 2.1.1. Team payoffs and Nash equilibrium strategies

For a collection of competing teams ${\left\{f_{k}\right\}}_{k=1}^{n}$ in the bounded measurable and continuous games, we define the payoff to strategy *f*_*k*_ by assessing the cumulative wins and losses of all individuals

2.3 $$E(f_{k};f_{1},\ldots ,f_{k-1},f_{k+1},\ldots ,f_{n})=\sum_{\ell \neq k}\int_{-\infty }^{\infty }f_{k}(x)\left[\int_{-\infty }^{x}f_{\ell }(t)\;dt-\int_{x}^{\infty }f_{\ell }(t)\;dt\right]dx.$$

For the discrete game, the payoffs are derived and defined analogously, so for a collection of competing teams, the payoff to strategy *A*_*k*_ is

2.4 $$E(A_{k};A_{1},\ldots ,A_{k-1},A_{k+1},\ldots ,A_{n})=\sum_{\ell \neq k}\sum_{\;j\in Z}A_{k}(x_{j})\left[\sum_{i<j}A_{\ell }(x_{i})-\sum_{i>j}A_{\ell }(x_{i})\right].$$

Whenever a sum is empty, it is defined to be zero. As in [45,63], the definitions of these payoff functions correspond to individuals from the teams being randomly matched to compete. The way we have defined the payoffs, if a team doubles in size, then its payoff is multiplied by a factor of 2. In other words, if the team’s strategy simply changes by a positive scale factor, then its payoff changes by the same scale factor. We will show that this is irrelevant for determining the optimal strategies. However, for applications, one may wish to change this, for example by limiting the number of competitions based on the sizes of the competing teams. Since a team competes against all others, one could restrict the amount of competitions to be the smaller of (i) the size of the team and (ii) the cumulative size of all other competing teams. Consequently, to implement this, as in [63] one would multiply the payoff

$$E(f_{k};f_{1},\ldots ,f_{k-1},f_{k+1},\ldots ,f_{n}),$$

in the bounded measurable and continuous games (2.3) by the factor

$$\frac{\min\{\|f_{k}{\|}_{L^{1}},\sum_{\;j\neq k}\|f_{j}{\|}_{L^{1}}\}}{\|f_{k}{\|}_{L^{1}}\sum_{\;j\neq k}\|f_{j}{\|}_{L^{1}}}.$$

In the discrete game, one could implement the same consideration by multiplying *E* (*A*_*k*_; *A*_1_, …, *A*_*k*−1_, *A*_*k*+1_, …, *A*_*n*_) in (2.4) by the factor

$$\frac{\min\{|A_{k}|,\sum_{\;j\neq k}|A_{j}|\}}{\left|A_{k}|\sum_{\;j\neq k}|A_{j}\right|}.$$

In all cases, the factor in the numerator is the amount of competitions. The factor in the denominator corresponds to the probabilistic interpretation, so that the payoff is computed according to the probability that an individual from team *k* with a specific competitive ability competes with superior or inferior individuals from the other teams. It is straightforward to calculate that the payoffs satisfy a zero sum dynamic; see also [45,63]. So, one could allow internal competition within the team without affecting the payoffs and define the payoffs as done in [45] via

$$\begin{array}{rl} & E(f_{k};f_{1},\ldots ,f_{k-1},f_{k},f_{k+1},\ldots ,f_{n})\\ & =\frac{1}{\sum_{\;j=1}^{n}\|f_{j}{\|}_{L^{1}}}\sum_{\ell =1}^{n}\int_{-\infty }^{\infty }f_{k}(x)\left[\int_{-\infty }^{x}f_{\ell }(t)-\int_{x}^{\infty }f_{\ell }(t)\right]\;dx. \end{array}$$

Analogously in the discrete case, one could define the payoff *E* (*A*_*k*_; *A*_1_, …, *A*_*k*−1_, *A*_*k*_, *A*_*k*+1_, …, *A*_*n*_) to be

$$\frac{1}{\sum_{\;j=1}^{n}|A_{j}|}\sum_{\ell =1}^{n}\sum_{\;j\in Z}A_{k}(x_{j})\left[\sum_{i<j}A_{\ell }(x_{i})-\sum_{i>j}A_{\ell }(x_{i})\right].$$

These considerations may be useful for practical implementation. However, we will show below that to locate the optimal strategies, only the *sign* of the payoff matters (i.e. positive, negative or zero). For this reason, we will simply use the payoffs defined according to (2.3) and (2.4) for the sake of simplicity in the mathematical proofs. By *optimal strategy*, we mean a strategy that has non-negative payoff in competition with any other strategy. Simply put, this means that it always wins or breaks even. In [63], we called such a strategy a *non-exploitable strategy (nes)*. We will see that these strategies are connected to an important notion in game theory: an *equilibrium point*, also known as a *Nash equilibrium point* due to Nash’s proof of their existence [67]. An equilibrium point is a collection of strategies for all competing teams so that if any one team alone changes their strategy, their payoff does not increase.

**Definition 2.3.**

For *n* competing teams, an *equilibrium point* consists of *n* strategies for the *n* teams that satisfy the following condition: For each *k* = 1, …, *n*, if Team *k* changes its strategy but all other Teams ℓ for all ℓ ≠ *k* retain their strategies, then the payoff to Team *k* does not increase. That is to say, for all *k* = 1, …, *n*, we have in the bounded measurable and continuous games

$$E(f_{k};f_{1},\ldots ,f_{k-1},f_{k+1},\ldots ,f_{n})\geq E(g;f_{1},\ldots ,f_{k-1},f_{k+1},\ldots ,f_{n}),$$

for any strategy *g* of the same type (bounded measurable or continuous). In the discrete game of teams, similarly, the strategies must satisfy

$$E(A_{k};A_{1},\ldots ,A_{k-1},A_{k+1},\ldots ,A_{n})\geq E(B;A_{1},\ldots ,A_{k-1},A_{k+1},\ldots ,A_{n}),$$

for all *k* = 1, …, *n* and for all discrete strategies *B*. The strategies that compose an equilibrium point are known as *equilibrium strategies*.

In many contexts, it is reasonable to expect that the ‘best’ strategy for all players considered simultaneously are those in an equilibrium point [67]. Here, we will prove that equilibrium strategies are precisely those strategies which cannot be defeated; they have non-negative payoffs against *any* other strategy. For this reason, our results here identify all equilibrium strategies and all equilibrium points.

### 2.2. Teams characterized by equilibrium strategies are those with diverse individuals

**Figure 1.**
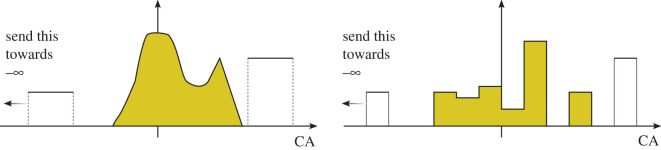
If there is no lower bound on the competitive abilities, then any strategy can be defeated. Starting with the yellow strategy, one constructs the *lowball* strategy by assigning competitive abilities below and above the support of the yellow strategy, such that more individuals are above the support, and fewer are individuals are below. Then, one sends the competitive abilities below towards −∞ until the MCA constraint is satisfied. Consequently, this *lowball* strategy could have *lower* mean competitive ability than the strategy it defeats.

In addition to developing this game theoretic model, which could be considered a result on its own, we identify all equilibrium points and equilibrium strategies for these games of teams. We first show that if one does not put a lower bound on the competitive abilities, then all strategies can be defeated. The idea is, given a strategy, since it has compact support, one can defeat it by assigning a few individuals competitive abilities towards −∞, thereby allowing the majority of individuals to have competitive abilities above the supremum of the compact support. This is somewhat unnatural because such a strategy could have mean competitive ability tending towards −∞, which would seem to be a non-optimal strategy. This *lowball* strategy is depicted in figure 1.

**Proposition 2.4.**

*If strategies may have any compact support, any strategy can be defeated*.

**Proof.**

Assume that a strategy *f* (bounded measurable or continuous) has compact support (as per definition of strategy). Then there exists an integer N∈N such that the support of *f* is contained in [−*N*, *N*]. In the bounded measurable game, let

2.5 $$g(x)=\left\{ \begin{array}{cc} 1,\; & -3N-2<x<-3N-1,\;\\ 2,\; & N+1<x<N+2,\;\\ 0,\; & \mathrm{for}\;\mathrm{all}\;\mathrm{other}\;\mathrm{values}\;\mathrm{of}\;x. \end{array}\right.$$

We calculate that MCA(g)=− N/3+ 1/2. Consequently, one may simply choose N∈N sufficiently large so that MCA(*g*) is less than the constraint value. We calculate that the payoff $E[g;f]=\|f{\|}_{L^{1}}>0$ by the definition of strategy. A similar construction can be used to construct a strategy *B* that defeats any given strategy *A* with compact support in the discrete game. In the continuous game, since continuous functions are dense in *L*^1^, one can simply approximate *g* by continuous functions and apply the dominated convergence theorem to obtain a continuous function $\tilde{g}$ such that $\mathrm{MCA}(\tilde{g})$ is less than the constraint value, and $E[\tilde{g},f]>\frac{1}{2}\|f{\|}_{L^{1}}>0$. ▪

The ‘lowballing’ strategy of placing competitive abilities below and above the support of a given strategy is depicted in figure 1. Since it is an artefact based on the ability to send competitive abilities towards ±∞, and moreover the defeating strategies may have *lower* MCA than the strategy they defeat, we suggest it is more natural to impose a lower bound on the range of CAs. With this assumption, there are equilibrium strategies.

**Theorem 2.5.**

*In the bounded measurable game, assume that all strategies have support contained in* [*a*, ∞), *and that the* MCA *constraint value* *C* > *a*. *A strategy is an equilibrium strategy if and only if it is almost everywhere equal to*

$$\left\{ \begin{array}{cc} c=\mathrm{constant}>0\; & on\;[a,2C-a]\;\\ 0\; & on\;(2C-a,\infty ].\; \end{array}\right.$$

*If one assumes that all strategies have support contained in* [*a*, *b*] *for fixed* −∞ < *a* < *b* < ∞, *then there are equilibrium strategies if and only if the constraint value* *C* ∈ (*a*, ((*b* + *a*)/2)], *and they are identical to those given above. Any collection of equilibrium strategies is an equilibrium point, and conversely, every equilibrium point* *comprises these equilibrium strategies. The sum of two or more equilibrium strategies is an equilibrium strategy. Equilibrium strategies have non-negative payoff in competition with any number of other strategies as long as those strategies are subject to the same constraint*.

**Remark 2.6.**

If the competitive ability values are uniformly bounded below by a∈R, then the MCA constraint value must be greater than or equal to *a*, otherwise there are no strategies. If the constraint value *C* = *a*, then the only strategies which satisfy definition 2.1 are almost everywhere equal to zero, because they may only be supported at *C* = *a*. This is not particularly interesting. If the competitive ability values are uniformly bounded below and above, they are contained within a fixed interval [*a*, *b*]. If the MCA constraint value is larger than the midpoint of this interval, then equilibrium strategies would be the same as in the case in which the competitive ability values are contained in [*a*, ∞), but this is impossible if 2*C* − *a* > *b* because the equilibrium strategies do not ‘fit’ within the prescribed interval [*a*, *b*]. This could be understood as an artefact of choosing an interval that is too small relative to the MCA value and remedied by considering the competition on the larger interval [*a*, 2*C* − *a*].

**Figure 2.**
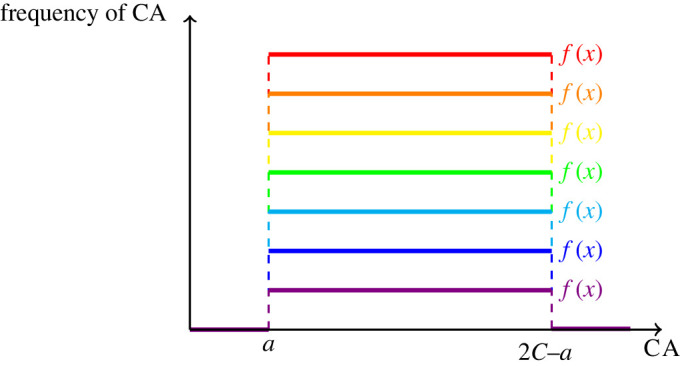
These are examples of equilibrium strategies in the bounded measurable game of teams. There are infinitely many equilibrium strategies, because any function that is constant and positive on [*a*, 2*C* − *a*] and zero elsewhere is an equilibrium strategy. Here, *a* is their minimum CA value, and *C* is the value of the constraint on the MCA. Consequently, teams characterized by an equilibrium strategy span the whole range of diverse competitive abilities from the minimum value, *a* up to twice the constraint value minus *a*. Equivalently, a team characterized by an equilibrium strategy allocates resources evenly across all team members, centred around the constraint value.

**Figure 3.**
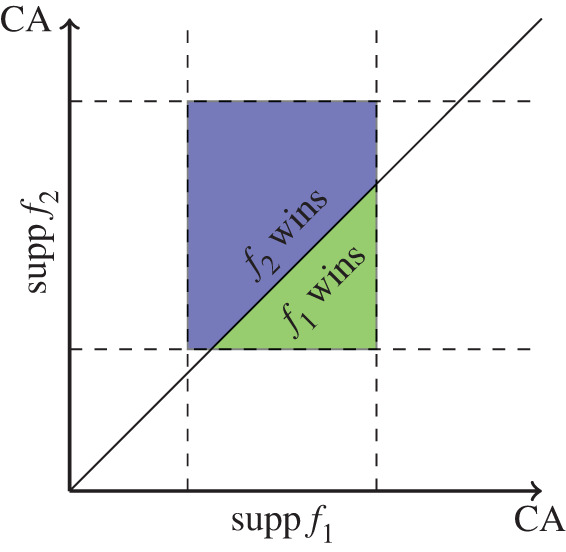
This is a visualization of the game between two competing teams showing the dependence on the teams’ competitive abilities and strategies. Competition occurs in the blue and green rectangle, where both teams have support. In the green area, team 1 is winning. In the blue area, team 2 is winning. The outcome of the game depends on how many individuals each team has in these areas, and this allocation of competitive abilities to the members of the team is determined by the team’s strategy.

A visualization of equilibrium strategies for the bounded measurable game is shown in figure 2, but we note that these are just finitely many examples of the infinitely many equilibrium strategies. In the case of two competing teams, a visualization of the game is shown in figure 3.

**Theorem 2.7.**

*In the continuous game, assume that all strategies have support contained in* [*a*, ∞) *for a fixed* a∈R. *Assume that the* MCA *constraint value* *C* ≥ *a*. *Then, there are no equilibrium strategies. If all strategies are supported in* [*a*, *b*] *for fixed real numbers* *a* < *b*, *and are only required to be continuous on* [*a*, *b*], *then there are equilibrium strategies if and only if the constraint value* *C* = (*b* + *a*)/2. *In this case, the equilibrium strategies are all constant positive functions on the interval* [*a*, *b*]. *Any collection of equilibrium strategies is an equilibrium point, and conversely, every equilibrium point* *comprises these equilibrium strategies. The sum of two or more equilibrium strategies is an equilibrium strategy. Equilibrium strategies have non-negative payoff in competition with any number of other strategies as long as those strategies are subject to the same constraint*.

**Remark 2.8.**

The obstruction to the existence of equilibrium strategies for the continuous game is that the functions that should be equilibrium strategies are those in the bounded measurable game. These strategies are not continuous on [*a*, ∞). If one considers continuous strategies only within a bounded interval, [*a*, *b*], and only requires strategies to be continuous on this interval, then precisely when the constraint value is the midpoint of this interval, equilibrium strategies exist.

**Theorem 2.9.**

*In the discrete case, assume that the set of competitive abilities is* ${\left\{x_{j}=\frac{j}{M}\right\}}_{\;j\geq a},$ *with constraint value* $C=\frac{k+a}{2M}$, *for integers* a∈Z *and* k>0. *If* *k* + *a* *is odd, then* *B* *is an equilibrium strategy if and only if it satisfies for some constant* *c* > 0,

$$\left\{ \begin{array}{cc} B(x_{\;j})=c,\; & a\leq j\leq k,\;\\ B(x_{\;j})=0,\; & k<j.\; \end{array}\right.$$

*If* *k* + *a* *is even, then* *B* *is an equilibrium strategy if and only if* MCA(*B*) = *C*, *and* *B*(*x*_2*j*+*a*_) = *B*(*x*_*a*_), *and* *B*(*x*_2*j*+*a*+1_) = *B*(*x*_*a*+1_) *for* *j* = 0, …, *k*, *with* *B*(*x*_*j*_) = 0 *for all* *j* > *k*. *If the set of competitive abilities is instead* ${\left\{x_{j}=\frac{j}{M}\right\}}_{a\leq j\leq b},$ *with constraint value* $C=\frac{k+a}{2M},$ *for integers* a<b∈Z *and* k>0, *then if the constraint value* *C* ≤ (*b* + *a*)/2(*M*), *equilibrium strategies are the same as those given above. In all cases, any collection of equilibrium strategies is an equilibrium point, and conversely, every equilibrium point* *comprises these equilibrium strategies. In all cases, the sum of two or more equilibrium strategies is an equilibrium strategy. Equilibrium strategies have non-negative payoff in competition with any number of other strategies as long as those strategies are subject to the same constraint*.

**Figure 4.**
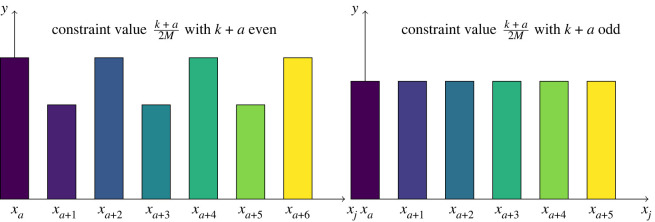
These are examples of equilibrium strategies in the discrete game of teams. There are infinitely many equilibrium strategies. The common feature they all share is that these strategies always span the whole range of diverse competitive abilities from *x*_*a*_ = *a*/*M* to 2*C* − *a*/*M*.

In the discrete game, there are also infinitely many equilibrium strategies with examples shown in figure 4. The equilibrium strategies are characterized by the distribution of competitive abilities spanning the range from *a*/*M* to 2*C* − (*a*/*M*), with *a*/*M* the minimum competitive ability value, and *C* the MCA constraint value. Any strategy that is not an equilibrium strategy can be defeated, in the sense that we provide a recipe in the proofs of our theorems to construct a strategy that will defeat any non-equilibrium strategy in competition. By contrast, equilibrium strategies can never be beaten. Equilibrium strategies always win or break even in competition with any number of strategies as long as those strategies satisfy the same MCA constraint.

## 3. Mathematical proofs

We begin by proving that the games are translation invariant in a certain sense. This allows us to reduce to the case in which all strategies are supported in [0, 1]. Next, we demonstrate a sufficient condition for a strategy to be an equilibrium strategy. Namely, it is sufficient that the payoff in competition with any other strategy is non-negative. We then prove that the equilibrium strategies given in theorems 2.5 and 2.9 satisfy this condition by explicitly computing the payoffs according to their definitions. Moreover, we determine all equilibrium strategies in the case of two competing teams. Finally, we complete the proof by demonstrating that the sufficient condition to be an equilibrium strategy is also necessary, so in fact, we locate all equilibrium strategies in this way.

### 3.1. Translation invariance

If there is a collection of competing teams, then there is a bounded closed interval that contains all of their supports. By possibly expanding the interval, we may assume that it is of the form [*a*, *b*], and that the constraint value *C* ∈ (*a*, *b*). The following lemma shows that it is equivalent to assume the interval is [0, 1].

**Lemma 3.1.**

*Assume that* *f* *and* *g* *are both non-negative bounded measurable functions whose supports are contained in an interval* [*a*, *b*] *for a bounded interval with* −∞ < *a* < *b* < ∞. *Define* ℓ = *b* − *a*, *and assume that* *C* ∈ [*a*, *b*]. *Let*

$$F(x)=\int_{a}^{x}f\;(t)\;dt,\;x\in [a,b],\;G(x)=\int_{a}^{x}g(t)\;dt.$$

*Assume that* *f* *and* *g* *are both not identically zero. The payoff is defined to be*

$$E[f;g]=\int_{a}^{b}f\;(x)\left(\int_{a}^{x}g(t)\;dt-\int_{x}^{b}g(t)\;dt\right)\;dx.$$

*Then, define*

$$\tilde{h}(t)=\ell h(t\ell +a),\;h\in \{f,g\},\;\tilde{C}=\frac{C-a}{\ell }.$$

*Then* *f* *and* *g* *satisfy the constraint*

3.1 $$\int_{a}^{b}xh(x)\;dx\leq C\int_{a}^{b}h(x)\;dx\;⟺\;\int_{0}^{1}t\tilde{h}(t)\;dt\leq \tilde{C}\int_{0}^{1}\tilde{h}(t)\;dt,\;h\in \{f,g\}.$$

*Moreover the payoffs to* $\tilde{f}$ *and* $\tilde{g}$ *satisfy*

$$E[f;g]=E[\tilde{f};\tilde{g}],\;E[g;f]=E[\tilde{g};\tilde{f}].$$

**Proof.**

Using the change of variables *x* = *t*ℓ + *a* and notation $\tilde{h}(t)\;:\;=\ell h(t\ell +a)$, we compute that the constraint on the interval [*a*, *b*] is

$$\begin{array}{rl} & 0\leq \int_{a}^{a+\ell }(C-x)h(x)\;dx=\int_{0}^{1}[C-\ell t-a]h(t\ell +a)\ell \;dt\\ & =\int_{0}^{1}\left[\frac{C}{\ell }-\frac{a}{\ell }-t\right]\ell^{2}h(t\ell +a)\;dt=\ell \int_{0}^{1}(\tilde{C}-t)\tilde{h}(t)\;dt. \end{array}$$

Since ℓ > 0, we may divide by it, obtaining that

$$0\leq \int_{a}^{a+\ell }(C-x)h(x)\;dx\;⟺\;0\leq \int_{0}^{1}(\tilde{C}-t)\tilde{h}(t)\;dt,$$

which proves (3.1). Next, we compute that

$$\tilde{G}(s)=\int_{0}^{s}\tilde{g}(t)\;dt=\int_{0}^{s}\ell g(t\ell +a)\;dt=\int_{a}^{s\ell +a}g(x)\;dx=G(s\ell +a).$$

Consequently with *x* = *t*ℓ + *a*,

$$\begin{array}{rl} & E[f;g]=\int_{a}^{b}f\;(x)\left[\int_{a}^{x}g(y)\;dy-\int_{x}^{b}g(y)\;dy\right]\;dx=\int_{a}^{b}f\;(x)[G(x)-(G(b)-G(x))]\;dx\\ & =\int_{a}^{b}f\;(x)[2G(x)-G(b)]\;dx=\int_{0}^{1}f\;(t\ell +a)[2G(t\ell +a)-G(b)]\ell \;dt\\ & =\int_{0}^{1}\tilde{f}\;(t)[2\tilde{G}(t)-\tilde{G}(1)]\;dt=E[\tilde{f};\tilde{g}]. \end{array}\;\text{■}$$

No generality is therefore lost by assuming the competitive abilities are contained in the interval [0, 1] for the bounded measurable game as well as the continuous game. The following lemma shows that the same is true for the discrete game.

**Lemma 3.2.**

*Assume that* *A* *and* *B* *are discrete strategies that define maps*

$$A,B\;:\;{\left\{x_{j}=a+\frac{\;j\ell }{M}\right\}}_{\;j\geq 0}\to [0,\infty ),\;\ell =b-a,\;-\infty <a<b<\infty ,$$

*such that*

$$|A|=\sum_{\;j\geq 0}A(x_{j})>0\;\mathrm{and}\;|B|=\sum_{\;j\geq 0}B(x_{j})>0.$$

*Assume that* *C* ∈ (*a*, *b*). *Then*,

$$\mathrm{MCA}(A)=\frac{1}{\left|A\right|}\sum_{\;j\geq 0}x_{j}A(x_{j})\leq C\;⟺\;\mathrm{MCA}(\tilde{A})\leq \tilde{C}=\frac{C-a}{\ell },$$

*for*

$$\tilde{A}\;:\;{\left\{\frac{j}{M}\right\}}_{\;j\geq 0}\to [0,\infty ),\;\tilde{A}\left(\frac{\;j}{M}\right)=A(x_{j}).$$

*Moreover, for*

$$E[A;B]=\sum_{\;j\geq 0}A(x_{j})\left(\sum_{i<j}B(x_{i})-\sum_{i>j}B(x_{i})\right),$$

*and* $\tilde{B}$ *defined analogously to* $\tilde{A}$, *we have*

$$E[A;B]=E[\tilde{A};\tilde{B}]\;\mathrm{and}\;E[B;A]=E[\tilde{B};\tilde{A}].$$

**Proof.**

Note that $\left|A|=|\tilde{A}\right|$ and $\left|B|=|\tilde{B}\right|$. Then

$$\begin{array}{rl} & \mathrm{MCA}(\tilde{A})=\frac{1}{\left|\tilde{A}\right|}\sum_{\;j\geq 0}\frac{j}{M}\tilde{A}\left(\frac{\;j}{M}\right)=\frac{1}{\left|A\right|}\sum_{\;j\geq 0}\frac{j}{M}A(x_{j})=\frac{1}{\left|A\right|}\sum_{\;j\geq 0}\frac{x_{j}-a}{\ell }A(x_{j})\\ & =\frac{\mathrm{MCA}(A)-a}{\ell }\leq \tilde{C}=\frac{C-a}{\ell }\;⟺\;\mathrm{MCA}(A)\leq C. \end{array}$$

Moreover,

$$\begin{array}{rl} & E[A;B]=\sum_{\;j\geq 0}A(x_{j})\left(\sum_{i<j}B(x_{i})-\sum_{i>j}B(x_{i})\right)\\ & =\sum_{\;j\geq 0}\tilde{A}\left(\frac{\;j}{M}\right)\left(\sum_{i<j}\tilde{B}\left(\frac{i}{M}\right)-\sum_{i>j}\tilde{B}\left(\frac{i}{M}\right)\right)=E[\tilde{A};\tilde{B}]. \end{array}\;\text{■}$$

**Remark 3.3.**

In all cases, the constraint value *C* > *a*. In the cases in which the competitive ability values are only assumed to be bounded from below, since all strategies are compactly supported, for any finite collection of competing strategies there is *R* > *a* so that their supports are all contained in [*a*, *R*]. Consequently, when the strategies are supported in [*a*, ∞), we may without loss of generality, assume *R* ≥ 2*C* − *a*. By the preceding lemmas, this is equivalent to analysing competition for strategies supported in [0, 1] with constraint value *C* ≤ 1/2. If the competitive ability values are instead subject to the same fixed lower *and* upper bounds, this reduces to analysing competition for strategies supported in [0, 1] with the possibility that the MCA constraint value may be larger than 1/2. We will therefore analyse these cases.

### 3.2. A sufficient condition for equilibrium strategies

Here, we demonstrate a sufficient condition for a collection of strategies to be an equilibrium point. This allows us to reduce to considering pairwise competition. Once we complete the analysis for pairwise competition, we will prove that the sufficient condition is also necessary and thereby identify all equilibrium strategies. We begin by computing for two competing strategies,

3.2 $$\begin{array}{rl} E[f;g] & =\int_{0}^{1}f\;(x)(2G(x)-G(1))\;dx\\ & =\left(F(1)G(1)-2\int_{0}^{1}F(x)g(x)\;dx\right)\\ & =\int_{0}^{1}g(x)(F(1)-2F(x))\;dx\\ & =-E[g;f]\;⟹\;E[f;g]+E[g;f]=0. \end{array}$$

This reflects the fact that each team collects all its winnings and pays all its losses to the competing teams, hence the total value across all teams remains constant. One could interpret this as competition for a limited amount of resources. We therefore have for a collection of competing teams

$$\sum_{k=1}^{n}E(f_{k};\ldots )=0,$$

where *E*(*f*_*k*_; …) indicates the payoff to strategy *f*_*k*_ competing against all others.

As shown in [45,63]

3.3 E[A;B]+E[B;A]=0 ⟹ E[B;A]=−E[A;B],

and similarly for a collection of competing teams,

$$\sum_{k=1}^{n}E(A_{k};\ldots )=0.$$

Above *E*(*A*_*k*_; …) indicates the payoff to strategy *A*_*k*_ competing against all others.

**Proposition 3.4.**

*Assume that a collection of strategies* (*f*_1_, …, *f*_*n*_) *for the bounded measurable and continuous games satisfies*

3.4 $$E(f_{k};f_{j})=0\forall j,k,\;E(f_{k};g)\geq 0\;\mathrm{for}\;\mathrm{any}\;\mathrm{strategy}\;g.$$

*Then* (*f*_1_, …, *f*_*n*_) *is an equilibrium point. The analogous statement holds for the discrete game*.

**Proof.**

Assume that a collection of strategies satisfies (3.4). Then it follows by the definition of the payoffs that for all *k* = 1, …, *n*,

$$E(f_{k};f_{1},\ldots ,f_{k-1},f_{k+1},\ldots ,f_{n})=0.$$

Moreover, by the zero sum dynamic, for any strategy *g* we have *E*(*g*; *f*_*k*_) ≤ 0, and so again by the definition of the payoffs, for all *k* = 1, …, *n*,

$$E(g;f_{1},\ldots ,f_{k-1},f_{k+1},\ldots ,f_{n})\leq 0=E(f_{k};f_{1},\ldots ,f_{k-1},f_{k+1},\ldots ,f_{n}).$$

This collection of strategies is therefore an equilibrium point. The argument for the discrete game is identical. ▪

### 3.3. The bounded measurable and continuous games of teams

**Proposition 3.5.**

*If* *C* = 1/2, *then a pair of functions that are positive and constant on* [0, 1] *and zero elsewhere is an equilibrium point for the bounded measurable game of teams*.

**Proof.**

For any *g* with *MCA*(*g*) ≤ *C* competing with

$$u(x)=\left\{ \begin{array}{cc} U(1),\; & 0\leq x\leq 1,\;\\ 0,\; & 1<x\; \end{array}\right.$$

and

$$\begin{array}{rl} & \frac{1}{U(1)G(1)}E[u;g]=\int_{0}^{1}\frac{1}{G(1)}\left(\int_{0}^{x}g(t)\;dt-\int_{x}^{1}g(t)dt\right)\;dx\\ & =\left(\int_{0}^{1}2\frac{G(x)}{G(1)}\;dx-1\right)=\left(1-\frac{2}{G(1)}\int_{0}^{1}xg(x)\;dx\right)=1-2\mathrm{MCA}(g)\geq 0. \end{array}$$

The inequality follows from the constraint (2.2) with *C* = 1/2. Moreover, if *g* is also positive and constant on [0, 1] and zero outside this interval, then MCA(*g*) = 1/2, and so we therefore have that *u* and *g* satisfy the necessary and sufficient conditions to be an equilibrium strategy. ▪

**Proposition 3.6.**

*Let* *f* *be a bounded measurable strategy subject to the constraint with* *C* = 1/2. *Assume that* *f* *is not constant on* [0, 1], *and that* *f* *is supported in* [0, 1]. *Then there exists a bounded measurable strategy* *g* *subject to the same constraint and supported in* [0, 1] *for which* *E*[*f*; *g*] < 0.

**Proof.**

If MCA(*f*) < 1/2 then a strategy *g*(*x*) that is positive and constant on [0, 1] and supported in this interval satisfies *E*[*f*; *g*] < 0. We may therefore henceforth assume MCA(*f*) = 1/2. Then,

$$-\int_{0}^{1}\frac{\;f\;(x)}{2}\;dx=-\frac{F(1)}{2}=-\int_{0}^{1}xf\;(x)\;dx$$

and

$$\begin{array}{rl} & E[f;g]=\int_{0}^{1}f\;(x)(2G(x)-G(1))\;dx=2G(1)\int_{0}^{1}f\;(x)\left(\frac{G(x)}{G(1)}-\frac{1}{2}\right)\;dx\\ & \;⟹\;E[f;g]=2G(1)\int_{0}^{1}f\;(x)\left(\frac{G(x)}{G(1)}-x\right)\;dx, \end{array}$$

and so similarly if MCA(*g*) = 1/2, we have

$$E[g;f]=2F(1)\int_{0}^{1}g(x)\left(\frac{F(x)}{F(1)}-x\right)\;dx.$$

Since *f* is not constant there exists *x* ∈ [0, 1] such that *F*(*x*) ≠ *xF*(1). Thus, the integrand above must assume both positive and negative values on sets of positive measure. We note that since $f\in L^{\infty }$, it follows that *F*(*x*) is continuous. Consequently, there is a non-empty open interval (*a*, *b*) ⊂ [0, 1], and a constant *R* > 0, such that

3.5 $$\frac{F(x)}{F(1)}-x>R\;\forall \;x\in [a,b].$$

If (*a*, *b*) is not fully contained in either (0, 1/2) or (1/2, 1), then we split (*a*, *b*) into smaller intervals, one of which is fully contained in either (0, 1/2) or (1/2, 1). We therefore assume without loss of generality that (*a*, *b*) is contained in either (0, 1/2) or (1/2, 1). First, assume (*a*, *b*) ⊂ (1/2, 1). Define for positive parameters *M*, *N* and *δ* < *a*,

3.6 $$g(x)=\left\{ \begin{array}{cc} M\; & x\in [0,\delta ]\;\\ N\; & x\in (a,b)\;\\ 0\; & \mathrm{otherwise}.\; \end{array}\right.$$

We will choose these parameters such that *g* is a bounded measurable strategy supported in [0, 1] with MCA(*g*) = 1/2, and for which *E*[*f*; *g*] < 0. To do this, we compute

$$G(1)=M\delta +N(b-a),\;\mathrm{MCA}(g)=\frac{M\delta^{2}/2+N(b^{2}-a^{2})/2}{M\delta +N(b-a)}.$$

To guarantee that MCA(*g*) = 1/2, we therefore require

3.7 $$\begin{array}{rl} & M\delta^{2}+N(b^{2}-a^{2})=M\delta +N(b-a)\\ & \;⟺\;M=\frac{N(b-a)(b+a-1)}{\delta (1-\delta )}. \end{array}$$

Since (*a*, *b*) ∈ (1/2, 1), *b* + *a* − 1 > 0, and so it is possible to choose *M*, *N* > 0 and 0 < *δ* < 1 so that this equation is satisfied. Then,

$$\begin{array}{rl} & E[g;f]=2F(1)\int_{0}^{1}g(x)\left(\frac{F(x)}{F(1)}-x\right)\;dx\\ & =2F(1)\left[\int_{0}^{\delta }M\left(\frac{F(x)}{F(1)}-x\right)\;dx+\int_{a}^{b}N\left(\frac{F(x)}{F(1)}-x\right)\;dx\right]\\ & \geq 2F(1)(-M\delta^{2}/2+NR(b-a))=F(1)\left(-\frac{N(b-a)(b+a-1)\delta }{1-\delta }+2NR(b-a)\right)\\ & =F(1)N(b-a)\left(2R-\frac{\delta (b+a-1)}{1-\delta }\right). \end{array}$$

Assume we choose *δ* ∈ (0, 1/2). Then since *b* + *a* − 1 ≤ 1, *E*[*g*; *f*] > 0 for any choice of δ∈(0,R)∩(0,1/2).

If (*a*, *b*) ⊂ (0, 1/2), define for a constant *δ* with 0 < *δ* < 1/2,

3.8 $$g(x)=\left\{ \begin{array}{cc} N\; & x\in (a,b)\;\\ M\; & x\in (1-\delta ,1]\;\\ 0\; & \mathrm{otherwise}.\; \end{array}\right.$$

Then

$$G(1)=N(b-a)+M\delta ,\;\mathrm{MCA}(g)=\frac{N(b^{2}-a^{2})/2+M(2\delta -\delta^{2})/2}{N(b-a)+M\delta }.$$

We therefore fix

$$\begin{array}{rl} & \mathrm{MCA}(g)=\frac{1}{2}\;⟹\;N(b^{2}-a^{2})+M(2\delta -\delta^{2})=N(b-a)+M\delta \\ & \;⟺\;M=\frac{N(b-a)(1-b-a)}{\delta (1-\delta )}. \end{array}$$

Noting that (*a*, *b*) ⊂ (0, 1/2), it is therefore possible to choose *N*, *M* > 0 and *δ* ∈ (0, 1) to satisfy this equality.

Then, we estimate

$$\begin{array}{rl} & \left|\frac{F(x)}{F(1)}-x\right|=\left|\frac{F(x)-F(1)+F(1)(1-x)}{F(1)}\right|\leq \frac{\left|F(x)-F(1)\right|}{F(1)}+|1-x|\\ & \leq \left(\frac{\|f{\|}_{\infty }}{F(1)}+1\right)(1-x),\;x\in (0,1), \end{array}$$

having used

$$|F(x)-F(1)|=F(1)-F(x)=\int_{x}^{1}f\;(x)\;dx\leq (1-x)\|f{\|}_{\infty }.$$

Hence

$$\begin{array}{rl} & E[g;f]=2F(1)\int_{0}^{1}g(x)\left(\frac{F(x)}{F(1)}-x\right)\;dx\\ & =2F(1)\left[\int_{a}^{b}N\left(\frac{F(x)}{F(1)}-x\right)\;dx+\int_{1-\delta }^{1}M\left(\frac{F(x)}{F(1)}-x\right)\;dx\right]\\ & \geq 2F(1)\left(NR(b-a)-M\delta^{2}\left(1+\frac{\|f{\|}_{\infty }}{F(1)}\right)\right)\\ & =2F(1)\left(NR(b-a)-\frac{N(b-a)(1-b-a)\delta }{\left(1-\delta \right)}\left(1+\frac{\|f{\|}_{\infty }}{F(1)}\right)\right)\\ & =2F(1)N(b-a)\left(R-\delta \frac{\left(1-b-a\right)}{1-\delta }\left(1+\frac{\|f{\|}_{\infty }}{F(1)}\right)\right). \end{array}$$

Since (*a*, *b*) ⊂ (0, 1/2), 0 < 1 − *b* − *a* < 1, so assuming that *δ* ∈ (0, 1/2), we obtain that *E*[*g*; *f*] > 0 for any

$$\delta \in \left(0,\frac{R}{2(1+\|f{\|}_{\infty }/F(1))}\right)⋂\left(0,\frac{1}{2}\right).\;\text{■}$$

**Corollary 3.7.**

*In the case when the constraint* *C* = 1/2, *all equilibrium strategies in the bounded measurable game of teams for functions supported in* [0, 1] *are positive constants on the unit interval, and conversely, all equilibrium points* *comprise positive constant functions*.

**Proof.**

A collection of equilibrium strategies (*f*_1_, …, *f*_*n*_) must satisfy

$$E[f_{k};f_{j}]=0,\;\forall j,k,\;E[f_{k};g]\geq 0$$

for any other strategy *g*. By the preceding proposition, the only strategies that satisfy these conditions are those in the statement of the corollary. ▪

**Corollary 3.8.**

*In the continuous game of teams restricted to a fixed interval* [*a*, *b*] *with constraint value* *C* = (*b* + *a*)/2 *all equilibrium strategies are positive constant functions on* [*a*, *b*].

**Proof.**

In ([45], theorem 1), we proved that all equilibrium strategies, if we consider *only* the unit interval with constraint value *C* = 1/2, are constant positive functions. By the translation invariance, this is equivalent to considering the continuous game on [*a*, *b*] with constraint value *C* = (*b* + *a*)/2. ▪

We assume next that the constraint value is in (0, 1/2).

**Theorem 3.9.**

*Assume that* *C* ∈ (0, 1/2). *Then all equilibrium strategies in the bounded measurable game for functions supported in* [0, 1] *comprise elements of* $L^{\infty }$ *that are almost everywhere equal to*

$$\left\{ \begin{array}{cc} 0\; & x\in [2C,1]\;\\ a\; & x\in [0,2C]\; \end{array}\right.,$$

*for some positive constant* *a*. *For the continuous game, there are no equilibrium strategies*.

**Proof.**

We compute that the payoff

$$\begin{array}{rl} & E[g;f]=\int_{0}^{1}g(x)\left(\int_{0}^{x}f\;(t)\;dt-\int_{x}^{1}f\;(t)\;dt\right)\;dx\\ & =\int_{0}^{1}g(x)(2F(x)-F(1))\;dx=2F(1)\int_{0}^{1}g(x)\left(\frac{F(x)}{F(1)}-\frac{1}{2}\right)\;dx, \end{array}$$

Then, insert

$$\frac{1}{2}\int_{0}^{1}g(x)\;dx=\frac{1}{2}G(1)=\frac{1}{2\;\mathrm{MCA}(g)}\int_{0}^{1}xg(x)\;dx.$$

We then find that

3.9 $$\frac{1}{2F(1)}E[g;f]=\int_{0}^{1}g(x)\left(\frac{F(x)}{F(1)}-\frac{x}{2\;\mathrm{MCA}(g)}\right)\;dx$$

and

3.10 $$\frac{1}{2G(1)}E[f;g]=\int_{0}^{1}f\;(x)\left(\frac{G(x)}{G(1)}-\frac{x}{2\;\mathrm{MCA}(f)}\right)\;dx.$$

*Case 1 in the bounded measurable game:* Assume that *f* is not identically zero on the interval (2*C*, 1]. Since we are working in $L^{\infty }$, functions that differ on sets of measure zero are identical as elements of $L^{\infty }$, so it is equivalent to assume that *f* is positive on a set of positive measure inside (2*C*, 1]. Consider the function

3.11 $$h(x)=\left\{ \begin{array}{cc} 1\; & x\in [0,2C],\;\\ 0\; & x\in (2C,1].\; \end{array}\right.$$

Then, as it is defined, *h* is a strategy. We note that for any strategy *g*,

$$\frac{1}{2G(1)}E[f;g]=\int_{0}^{1}f\;(x)\left(\frac{G(x)}{G(1)}-\frac{x}{2\;\mathrm{MCA}(f)}\right)\;dx\leq \int_{0}^{1}f\;(x)\left(\frac{G(x)}{G(1)}-\frac{x}{2\;C}\right)\;dx,$$

because

$$\mathrm{MCA}(f)\leq C\;⟹\;\frac{x}{\mathrm{MCA}(f)}\geq \frac{x}{C}.$$

We then compute that for

3.12 $$H(x)=\int_{0}^{x}h(t)\;dt$$

and

$$\begin{array}{rl} & \frac{1}{2H(1)}E[f;h]\leq \int_{0}^{1}f\;(x)\left(\frac{H(x)}{H(1)}-\frac{x}{2\;C}\right)\;dx\\ & =\int_{2C}^{1}f\;(x)\left(\frac{H(x)}{H(1)}-\frac{x}{2C}\right)\;dx=\int_{2C}^{1}f\;(x)\left(1-\frac{x}{2C}\right)\;dx<0, \end{array}$$

since (1 − (*x*/2*C*)) < 0 on (2*C*, 1], and *f* is non-zero on (2*C*, 1] and strictly positive on a set of positive measure by assumption.

*Case 2 in the bounded measurable game*: Assume that *f*(*x*) = 0 for *x* ∈ [2*C*, 1] (equivalently, *f* = 0 almost everywhere in [2*C*, 1] but since we work in $L^{\infty }$ this is equivalent). The proof in this case then reduces to the case in which the constraint value is 1/2, and the competitive abilities are selected from the range [0, 1] by lemma 3.1.

*Case 1 in the continuous game*: Assume that *f* is not identically zero on the interval (2*C*, 1]. In this case, we shall begin with a bounded measurable function that is discontinuous and approximate it by continuous functions. For the functions *h* and *H* defined in (3.11) and (3.12), respectively, we have computed in case 1 of the bounded measurable game that *E*[*f*; *h*] < 0. Since we require continuous functions, we define

$$h_{\varepsilon }(x)=\left\{ \begin{array}{cc} \frac{x}{\varepsilon }\; & x\in [0,\varepsilon ],\;\\ 1\; & x\in [\varepsilon ,2C-\varepsilon ],\;\\ -\frac{x}{\varepsilon }+\frac{2C}{\varepsilon },\; & x\in [2C-\varepsilon ,2C],\;\\ 0\; & x\in [2C,1].\; \end{array}\right.$$

**Figure 5.**
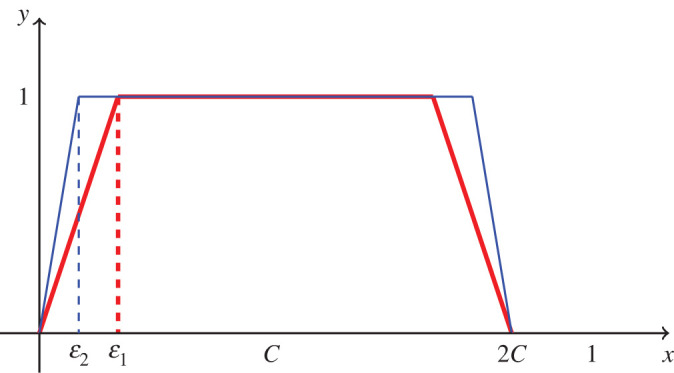
These are graphs of $h_{\varepsilon_{1}}$ and $h_{\varepsilon_{1}}$ with *ɛ*_1_ > *ɛ*_2_.

Let us note that *h*_*ɛ*_ is a continuous non-negative function, and MCA(*h*_*ɛ*_) = *C* since it is symmetric with respect to *C* on [0, 2*C*] and identically zero on [2*C*, 1]. These functions for different values of *ɛ* are shown in figure 5. Next, we note that *h*_*ɛ*_ → *h* pointwise almost everywhere on [0, 1] as *ɛ* → 0, therefore,

$$f\;(x)\left(\frac{H_{\varepsilon }(x)}{H_{\varepsilon }(1)}-\frac{x}{2C}\right)\to f\;(x)\left(\frac{H(x)}{H(1)}-\frac{x}{2C}\right),$$

pointwise almost everywhere on [0, 1] as *ɛ* → 0. The dominated convergence theorem and *E*[*f*; *h*] < 0 imply that

$$\int_{0}^{1}f\;(x)\left(\frac{H_{\varepsilon }(x)}{H_{\varepsilon }(1)}-\frac{x}{2C}\right)\;dx<0\;⟹\;E[f;h_{\varepsilon }]<0,$$

for sufficiently small *ɛ* > 0.

*Case 2 in the continuous game:* Assume that *f*(*x*) = 0 for *x* ∈ [2*C*, 1]. We then consider

$$\tilde{f}\;(t)=f\;(2Ct),\;t\in [0,1].$$

Since *f* is continuous on [0, 1], *f* cannot be a positive constant on [0, 2*C*], and therefore *f*(2*Ct*) is not equal to a positive constant for *t* ∈ [0, 1]. The proof in this case follows from lemma 3.1 and theorem 1 in [45]. ▪

**Corollary 3.10.**

*In the continuous game of teams with a fixed lower bound for the competitive ability but no fixed upper bound, there are no equilibrium strategies*.

**Proof.**

If we require the strategies to be continuous on [*a*, ∞), then by the translation invariance, this reduces to considering the interval [0, 1] with MCA constraint value *C* ∈ (0, 1/2]. If *C* < 1/2, then there are no continuous equilibrium strategies by theorem 3.9. If *C* = 1/2, then functions that are constant and positive on [0, 1] are equilibrium strategies (ignoring the exterior of this interval). By contradiction, if a continuous function on [*a*, ∞) were an equilibrium strategy, then its translation to [0, 1] should be positive and constant on this entire interval. However, translating back to [*a*, ∞), this function would need to be both positive and constant on [*a*, 2*C* − *a*] *and* continuous on [*a*, ∞). That is impossible. So in this case, there are also no equilibrium strategies. ▪

**Theorem 3.11.**

*Assume that* *C* ∈ (1/2, 1]. *Then there are no equilibrium strategies* *either for the bounded measurable game of teams* *or for the continuous game of teams with competitive abilities contained in* [0, 1].

**Proof.**

Assume that *f* is bounded and measurable, noting that if *f* is continuous then this is immediately the case. We will construct a continuous (and therefore also bounded and measurable) function *g* subject to the same constraint such that *E*[*f*; *g*] < 0. For

$$F(x)=\int_{0}^{x}f\;(t)\;dt,$$

since *f* is bounded and measurable, *F* is continuous. Since *C* > 1/2,

$${\frac{F(x)}{F(1)}-\frac{x}{2C}|}_{x=1}=\frac{F(1)}{F(1)}-\frac{1}{2C}=1-\frac{1}{2C}>0.$$

The continuity of *F* ensures that there is a *δ* ∈ (0, 1) such that

3.13 $$\frac{F(x)}{F(1)}-\frac{x}{2C}>0\;\forall x\in [1-\delta ,1].$$

**Lemma 3.12.**

*If*

$$g(x)=\left\{ \begin{array}{cc} M^{2}\left(\frac{1}{M}-x\right),\; & x\in \left[0,\frac{1}{M}\right]\;\\ 0,\; & x\in \left[\frac{1}{M},1-\frac{1}{M}\right]\;\\ B\left(x-1+\frac{1}{M}\right),\; & x\in \left[1-\frac{1}{M},1\right]\; \end{array}\right.$$

*then*

$$G(1)=\frac{1}{2}+\frac{B}{2M^{2}}\;\mathrm{and}\;\mathrm{MCA}(g)=\frac{M^{2}+B(3M-1)}{3M(B+M^{2})}.$$

**Proof of lemma 3.12.**

The formulae follow from direct computation. We will split the integral computations into the two intervals [0, 1/*M*] and [1 − 1/*M*, 1], since

$$G(1)=\int_{0}^{1/M}g(x)\;dx+\int_{1-1/M}^{1}g(x)\;dx$$

and

$$\mathrm{MCA}(g)=\frac{1}{G(1)}\left(\int_{0}^{1/M}xg(x)\;dx+\int_{1-1/M}^{1}xg(x)\;dx\right).$$

We find

$$\begin{array}{rl} & \int_{0}^{1/M}xg(x)\;dx=\int_{0}^{1/M}(Mx-M^{2}x^{2})\;dx\\ & =M{\frac{x^{2}}{2}|}_{0}^{1/M}-M^{2}{\frac{x^{3}}{3}|}_{0}^{1/M}=\frac{1}{2M}-\frac{1}{3M}=\frac{1}{6M} \end{array}$$

and

$$\begin{array}{rl} & \int_{1-1/M}^{1}\left(Bx^{2}-\left[1-\frac{1}{M}\right]Bx\right)\;dx=B{\frac{x^{3}}{3}|}_{1-1/M}^{1}-\left[1-\frac{1}{M}\right]B{\frac{x^{2}}{2}|}_{1-1/M}^{1}\\ & =B\left(\frac{1}{3}-\frac{{\left(1-1/M\right)}^{3}}{3}\right)-\left(1-\frac{1}{M}\right)B\left(\frac{1}{2}-\frac{{\left(1-1/M\right)}^{2}}{2}\right)\\ & =\frac{B}{3}\left(\frac{3M-3M^{2}+1}{M^{3}}\right)-\frac{B}{2}\left(\frac{M-1}{M}\right)\left(\frac{2M-1}{2}\right)\\ & =\frac{B}{6}\left(\frac{6M^{2}-6M+2-3(2M^{2}-3M+3)}{M^{3}}\right)=\frac{B}{6}\left(\frac{3M-1}{M^{3}}\right). \end{array}$$

Also,

$$\int_{0}^{1/M}g(x)\;dx=\int_{0}^{1/M}(M-M^{2}x)\;dx=1-M^{2}\frac{1}{2M^{2}}=\frac{1}{2}$$

and

$$\begin{array}{rl} & \int_{1-1/M}^{1}g(x)\;dx=\int_{1-1/M}^{1}\left(Bx-\left[1-\frac{1}{M}\right]B\right)\;dx\\ & =B\frac{1-{\left(1-1/M\right)}^{2}}{2}-\left[1-\frac{1}{M}\right]B\left(1-\left[1-\frac{1}{M}\right]\right)\\ & =B\left(\frac{1}{2}{\left[1-\frac{1}{M}\right]}^{2}-\left[1-\frac{1}{M}\right]+\frac{1}{2}\right)\\ & =\frac{B}{2}{\left(\left[1-\frac{1}{M}\right]-1\right)}^{2}=\frac{B}{2M^{2}}. \end{array}$$

Thus,

$$G(1)=\frac{B}{2M^{2}}+\frac{1}{2}$$

and

$$\int_{0}^{1}xg(x)\;dx=\frac{1}{6M}+\frac{B}{6}\left(\frac{3M-1}{M^{3}}\right).$$

The formula for MCA(*g*) follows directly by dividing by *G*(1). ▪

We proceed by taking *g* as in lemma 3.12 and solving for *B* so that MCA(*g*) = *C*

3.14 $$\frac{M^{2}+B(3M-1)}{3M(B+M^{2})}=C\;⟺\;B=\frac{M^{2}(3CM-1)}{3M(1-C)-1}.$$

Since *B* should be positive, we must choose the parameter *M* sufficiently large, so we assume that it is chosen to satisfy

3.15 $$M>\max\left\{\frac{1}{3C},\frac{1}{3(1-C)}\right\}.$$

Denote

$$I_{1}=\int_{0}^{1/M}\left(\frac{F(x)}{F(1)}-\frac{x}{2C}\right)g(x)\;dx\;\mathrm{and}\;I_{2}=\int_{1-1/M}^{1}\left(\frac{F(x)}{F(1)}-\frac{x}{2C}\right)g(x)\;dx.$$

We estimate using the definition of *F*,

$$\left|\frac{F(x)}{F(1)}-\frac{x}{2C}\right|\leq x\frac{\|f{\|}_{\infty }}{F(1)}+\frac{x}{2C}\leq \frac{\|f{\|}_{\infty }}{MF(1)}+\frac{1}{2MC},\;\forall x\in [0,1/M].$$

Above ‖*f*‖_∞_ is the supremum norm of *f* which is finite by assumption. Since 0 ≤ *g*(*x*) ≤ *M* for all *x* ∈ [0, 1/*M*], we therefore estimate

3.16 $$|I_{1}|\leq \int_{0}^{1/M}M\left(\frac{\|f{\|}_{\infty }}{MF(1)}+\frac{1}{2MC}\right)\;dx=\frac{1}{M}\left(\frac{\|f{\|}_{\infty }}{F(1)}+\frac{1}{2C}\right).$$

We would like to obtain an estimate for *I*_2_ from below. In addition to the conditions (3.15), let us further assume that

3.17 $$M>\frac{1}{\delta }=\Rightarrow \frac{1}{M}<\delta .$$

By the inequality (3.13),

$$\exists \gamma >0\;:\;\frac{F(t)}{F(1)}-\frac{t}{2C}\geq \gamma \;\forall t\in [1-1/M,1].$$

We therefore obtain the estimate

$$I_{2}\geq \int_{1-1/M}^{1}\gamma g(x)\;dx=\int_{1-1/M}^{1}\gamma B\left(x-1+\frac{1}{M}\right)\;dx=\frac{\gamma }{2}\frac{B}{M^{2}}.$$

This leads to the estimate

$$I_{1}+I_{2}\geq I_{2}-|I_{1}|\geq \frac{\gamma }{2}\frac{B}{M^{2}}-\frac{1}{M}\left(\frac{\|f{\|}_{\infty }}{F(1)}+\frac{1}{2C}\right).$$

Therefore, in order to ensure that *I*_1_ + *I*_2_ > 0, recalling the expression for *B* in (3.14), we require

3.18 $$\begin{array}{rl} & \frac{\gamma }{2M^{2}}\frac{M^{2}(3CM-1)}{\left(3M(1-C)-1\right)}-\frac{1}{M}\left(\frac{\|f{\|}_{\infty }}{F(1)}+\frac{1}{2C}\right)>0\\ & \;⟺\;\frac{M(3CM-1)}{3M(1-C)-1}>\frac{2}{\gamma }\left(\frac{\|f{\|}_{\infty }}{F(1)}+\frac{1}{2C}\right). \end{array}$$

The left side of (3.18) tends to infinity with *M*, whereas the right side is fixed and bounded. Consequently, it is possible to choose *M* sufficiently large so that (3.15), (3.17) and (3.18) are all satisfied, and we thereby obtain

$$\begin{array}{rl} & \frac{1}{2F(1)}E[g,f]=I_{1}+I_{2}\geq I_{2}-|I_{1}|\\ & \geq \frac{\gamma }{2}\frac{3CM-1}{(3M(1-C)-1}-\frac{1}{M}\left(\frac{\|f{\|}_{\infty }}{F(1)}+\frac{1}{2C}\right)>0\\ & \;⟹\;E[g,f]>0. \end{array}\;\text{■}$$

**Proposition 3.13.**

*Assume that a collection of strategies* (*f*_1_, …, *f*_*n*_) *for the bounded measurable or continuous game is an equilibrium point. Then they satisfy*

$$E(f_{k};f_{j})=0\forall j,k,\;E(f_{k};g)\geq 0\;\mathrm{for}\;\mathrm{any}\;\mathrm{strategy}\;g.$$

*Equivalently, each of* *f*_*k*_ *is an equilibrium strategy for the two-player game*.

**Proof.**

Assume that (*f*_1_, …, *f*_*n*_) is an equilibrium point. Then by definition of equilibrium point

3.19 $$E(f_{k};f_{1},\ldots ,f_{k-1},f_{k+1},\ldots ,f_{n})\geq E\left(\sum_{\ell \neq k}f_{\ell };f_{1},\ldots ,f_{k-1},f_{k+1},\ldots ,f_{n}\right)=0.$$

Above, $\sum_{\ell \neq k}f_{\ell }$ is the strategy obtained by summing the strategies *f*_ℓ_ for all ℓ ≠ *k*. Note that this is also a strategy. The above inequality holds for all *k* = 1, …, *n*. By the zero sum dynamic

E[f;g]+E[g;f]=0,

for any arbitrary two strategies *f* and *g*. By induction, we will show that for any collection of strategies ${\left\{g_{k}\right\}}_{k=1}^{m}$, we have

$$\sum_{k=1}^{m}E(g_{k};g_{1},\ldots ,g_{k-1},g_{k+1},\ldots g_{m})=0.$$

For *m* = 2, this is true. Assume this also holds for some *m* ≥ 2. Then, by the definition of our payoff functions

$$\begin{array}{rl} & \sum_{k=1}^{m+1}E(g_{k};g_{1},\ldots ,g_{k-1},g_{k+1},\ldots g_{m+1})=\sum_{k=1}^{m+1}\sum_{\ell \neq k}E(g_{k};g_{\ell })\\ & =\sum_{k=1}^{m}\sum_{\ell \notin \{k,m+1\}}E(g_{k};g_{\ell })+\sum_{\ell =1}^{m}E(g_{m+1};g_{\ell })+\sum_{\ell =1}^{m}E(g_{\ell };g_{m+1})=0. \end{array}$$

Above we have used the induction assumption and the fact that *E*(*g*_*m*+1_; *g*_ℓ_) + *E*(*g*_ℓ_; *g*_*m*+1_) = 0 for each ℓ. Applying this calculation to (*f*_1_, …, *f*_*n*_), we therefore have

$$\sum_{k=1}^{n}E(f_{k};f_{1},\ldots ,f_{k-1},f_{k+1},\ldots ,f_{n})=0.$$

Since each summand is non-negative by (3.19), they must all vanish. Consequently, for any strategy *g*, by definition of equilibrium strategy,

3.20 $$E(g;f_{1},\ldots ,f_{k-1},f_{k+1},\ldots ,f_{n})\leq 0=E(f_{k};f_{1},\ldots ,f_{k-1},f_{k+1},\ldots ,f_{n}),$$

and this holds for all *k* = 1, …, *n*. We therefore have for the particular choice *g* = *f*_*j*_ for some fixed *j* that

$$E\left(\;f_{j};\sum_{\ell \neq k}f_{\ell }\right)\leq 0\;\forall k,\;⟹\;\sum_{k=1}^{n}E\left(\;f_{j};\sum_{\ell \neq k}f_{\ell }\right)\leq 0.$$

We compute using the definition of the payoffs and the zero-sum dynamic

$$\begin{array}{rl} & 0\geq \sum_{k=1}^{n}E\left(\;f_{j};\sum_{\ell \neq k}f_{\ell }\right)=\sum_{k=1}^{n}\sum_{\ell \neq k}E(f_{j};f_{\ell })=\sum_{k=1}^{n}(n-1)E(f_{j};f_{k})\\ & =(n-1)\sum_{k\neq j}E(f_{j};f_{k})=(n-1)E(f_{j};f_{1},\ldots ,f_{\;j-1},f_{\;j+1},\ldots ,f_{n})\\ & \;⟹\;E(f_{j};f_{1},\ldots ,f_{\;j-1},f_{\;j+1},\ldots ,f_{n})\leq 0. \end{array}$$

Since this last inequality has been shown to be an equality, each of the summands must vanish, showing that

$$E\left(\;f_{j};\sum_{\ell \neq k}f_{\ell }\right)=0,\;\forall j,k.$$

Combining

$$E(f_{j};f_{1},\ldots ,f_{\;j-1},f_{\;j+1},\ldots ,f_{n})=0\;\mathrm{and}\;E\left(\;f_{j};\sum_{\ell \neq k}f_{\ell }\right)=0\;⟹\;E(f_{j};f_{k})=0,$$

for all *j* and *k*. Consider for a moment the case in which there are only two competing strategies. Then a necessary and sufficient condition for (*f*, *h*) to be an equilibrium point is that

3.21 E(f;h)=0=E(h;f),E(f;g)≥0,E(h;g)≥0, for all strategies g.

Moreover, having identified all equilibrium strategies in the two-player game, it follows that if *f* − *h* ≥ 0 and is not identically zero, then *f* − *h* is also an equilibrium strategy. Define

$$g_{k}=\sum_{\ell \neq k}f_{\ell }=\Rightarrow E(g;g_{k})\leq E(f_{k};g_{k})=0\;\mathrm{for}\;\mathrm{all}\;\mathrm{strategies}\;g,$$

having used (3.20). We also have

$$E(g_{k};g_{j})=0\;\forall j,k.$$

Consequently, each *g*_*k*_ is an equilibrium strategy for the two-player game. By linearity, the sum of two equilibrium strategies is again an equilibrium strategy. We therefore have

$$\sum_{k=1}^{n}g_{k}=(n-1)\sum_{k=1}^{n}f_{k},$$

is an equilibrium strategy. A non-zero scalar multiple of an equilibrium strategy is again an equilibrium strategy by linearity, hence

$$\sum_{k=1}^{n}f_{k},$$

is an equilibrium strategy. Then since *f*_*k*_ is not identically zero by definition of strategy,

$$\sum_{\ell =1}^{n}f_{\ell }-g_{k}=f_{k},$$

is an equilibrium strategy for the two-player game, for each *k* = 1, …, *n*. ▪

### 3.4. The discrete game of teams

As noted by the translation invariance, we may assume that all competitive ability values are contained in [0, 1]. In the case where the constraint value *C* = 1/2, we have found all equilibrium strategies in ([45], theorem 1). We summarize the results obtained in [45] that determine all equilibrium strategies for the constraint value *C* = 1/2.

**Theorem 3.14 (theorem 1 of [45]).**

*In the case when* *M* *is odd, and the constraint value* *C* = 1/2, *then all equilibrium strategies supported in*

$${\left\{\frac{j}{M}\right\}}_{\;j=0}^{M},$$

*are uniform strategies. A uniform strategy* *U* *satisfies* *U*(*x*_*i*_) = *a* *for all* 0 ≤ *i* ≤ *M*, *for some constant* *a* > 0. *In the case when* *M* *is even, then all equilibrium strategies are those* *A* *which have* |*A*| > 0, MCA(A)=1/2 *and furthermore satisfy*

$$A(x_{2j})=A(x_{0}),\;A(x_{2j+1})=A(x_{1}),\;\forall j\in \left\{0,1,\ldots ,\frac{M}{2}\right\}.$$

Next, we assume that the constraint value *C* < 1/2 and is similar to the case in which *C* = 1/2, namely the constraint value satisfies

$$C=\frac{\;j_{C}}{M}\;\mathrm{or}\;C=\frac{2j_{C}+1}{2M}.$$

The equilibrium strategies in this case are of two types, analogous to those in the case when *C* = 1/2 and *M* is either odd or even.

**Theorem 3.15.**

*If the* MCA *constraint is for* *C* = *j*_*C*_/*M* < 1/2*,* *then all equilibrium strategies are those with* MCA = *C* *that are of the form*

$$A(x_{2k})=\left\{ \begin{array}{cc} a,\; & 0\leq k\leq j_{C},\;\\ 0\; & k\geq j_{C}+1,\; \end{array}\right.\;A(x_{2k+1})=\left\{ \begin{array}{cc} b,\; & 0\leq k\leq j_{C}-1,\;\\ 0,\; & k\geq j_{C}.\; \end{array}\right.$$

*If the* MCA *constraint is for* *C* = ((2*j*_*C*_ + 1)/2*M*) < 1/2, *then all equilibrium strategies are of the form*

$$A(x_{k})=\left\{ \begin{array}{cc} c,\; & 0\leq k\leq 2j_{C}+1,\;\\ 0,\; & k\geq 2j_{C}+2,\; \end{array}\right.$$

*for any constant* *c* > 0.

**Proof.**

If *C* = *j*_*C*_/*M*, we define

$$A(x_{k})=\left\{ \begin{array}{cc} 1,\; & k\in \{0,1,2,\ldots ,2j_{C}\}\;\\ 0,\; & k>2j_{C}.\; \end{array}\right.$$

Then |*A*| = 2*j*_*C*_ + 1, and MCA(*A*) = *C*. We compute *E*[*A*; *B*] for competition against a strategy *B* subject to the same constraint

$$\begin{array}{rl} & E[A;B]=\sum_{k=0}^{2j_{C}}\left(\sum_{i=0}^{k-1}B(x_{i})-\sum_{i=k+1}^{M}B(x_{i})\right)\\ & =\sum_{k=0}^{2j_{C}}\left(2\sum_{i=0}^{k-1}B(x_{i})+B(x_{k})-|B|\right)\\ & =2\sum_{k=0}^{2j_{C}}\sum_{i=0}^{k-1}B(x_{i})+\sum_{k=0}^{2j_{C}}B(x_{k})-(2j_{C}+1)|B|\\ & =2\sum_{k=0}^{2j_{C}}(2j_{C}-k)B(x_{k})+\sum_{k=0}^{2j_{C}}B(x_{k})-(2j_{C}+1)|B|\\ & =2\sum_{k=0}^{2j_{C}}(j_{C}-k)B(x_{k})+(2j_{C}+1)\sum_{k=0}^{2j_{C}}B(x_{k})-(2j_{C}+1)\sum_{k=0}^{M}B(x_{k})\\ & =2\sum_{k=0}^{2j_{C}}(j_{C}-k)B(x_{k})-(2j_{C}+1)\sum_{k=2j_{C}+1}^{M}B(x_{k}). \end{array}$$

Since *B* is subject to the constraint

$$\mathrm{MCA}(B)=\frac{\sum_{k=0}^{M}\frac{k}{M}B(x_{k})}{\sum_{k=0}^{M}B(x_{k})}\leq C=\frac{\;j_{C}}{M}\;⟺\;\sum_{k=0}^{M}(k-j_{C})B(x_{k})\leq 0,$$

it follows that

3.22 $$\sum_{k=2j_{C}+1}^{M}(k-j_{C})B(x_{k})\leq \sum_{k=0}^{2j_{C}}(j_{C}-k)B(x_{k}).$$

Thus,

3.23 $$\begin{array}{rl} & E[A;B]\geq 2\sum_{k=2j_{C}+1}^{M}(k-j_{C})B(x_{k})-(2j_{C}+1)\sum_{k=2j_{C}+1}^{M}B(x_{k})\\ & =\sum_{k=2j_{C}+1}^{M}(2k-4j_{C}-1)B(x_{k})\geq 0. \end{array}$$

Since 2*k* − 4*j*_*C*_ − 1 ≥ 0 for all *k* ≥ 2*j*_*C*_ + 1, and *B*(*x*_*k*_) ≥ 0 for all *k*, equality holds in (3.23) if and only if *B*(*x*_*k*_) = 0 for all *k* > 2*j*_*C*_. If this is not the case, then *E*[*A*; *B*] > 0, and therefore *A* defeats *B*. We note that the same holds for any other team that has positive identical values at *x*_*k*_ for *k* = 0, …, 2*j*_*C*_ and zero at all other *x*_*k*_. Consequently, it suffices to consider the problem for the interval [0, 2*j*_*C*_/*M*] with competitive abilities

$$0<\frac{1}{M}<\ldots <\frac{2j_{C}}{M},\;C=\frac{\;j_{C}}{M}.$$

As shown in lemma 3.2, this problem is equivalent to the case in which the constraint is equal to 1/2, and *M* is even.

Now assume that the constraint value is of the second type, i.e. *C* = (2*j*_*C*_ + 1)/2*M*. Therefore,

$$\frac{1}{\left|B\right|}\sum_{k=1}^{M}B(x_{k})\frac{k}{M}\leq \frac{2j_{C}+1}{2M}\;⟺\;\sum_{k=0}^{M}kB(x_{k})\leq \frac{2j_{C}+1}{2}|B|=\left(\;j_{C}+\frac{1}{2}\right)\sum_{k=0}^{M}B(x_{k}).$$

Hence, the MCA constraint admits the following reformulation:

$$\sum_{k=0}^{M}\left(k-j_{C}-\frac{1}{2}\right)B(x_{k})\leq 0.$$

Thus,

3.24 $$\sum_{k=2j_{C}+2}^{M}\left(k-j_{C}-\frac{1}{2}\right)B(x_{k})\leq \sum_{k=0}^{2j_{C}+1}\left(\;j_{C}+\frac{1}{2}-k\right)B(x_{k}).$$

We define the strategy *A* such that

$$A(x_{k})=\left\{ \begin{array}{cc} 1,\; & k\in \{0,1,2,\ldots ,2j_{C}+1\}\;\\ 0,\; & k>2j_{C}+1.\; \end{array}\right.$$

Then |*A*| = 2*j*_*C*_ + 2, and MCA(*A*) = *C* = (*j*_*C*_/*M*) + (1/2*M*). The payoff

$$\begin{array}{rl} E[A;B] & =\sum_{k=0}^{2j_{C}+1}\left(2\sum_{i=0}^{k-1}B(x_{i})+B(x_{k})-\sum_{i=0}^{M}B(x_{i})\right)\\ & =2\sum_{k=0}^{2j_{C}+1}(2j_{C}+1-k)B(x_{k})+\sum_{k=0}^{2j_{C}+1}B(x_{k})-(2j_{C}+2)\sum_{k=0}^{M}B(x_{k})\\ & =\sum_{k=0}^{2j_{C}+1}(2j_{C}+1-2k)B(x_{k})-(2j_{C}+2)\sum_{k=2j_{C}+2}^{M}B(x_{k}). \end{array}$$

Using the MCA constraint and (3.24), we find

$$\begin{array}{rl} E[A;B] & \geq \sum_{k=2j_{C}+2}^{M}(2k-1-2j_{C})B(x_{k})-(2j_{C}+2)\sum_{k=2j_{C}+2}^{M}B(x_{k})\\ & =\sum_{k=2j_{C}+2}^{M}(2k-3-4j_{C})B(x_{k})\geq 0. \end{array}$$

Above, we use the facts that *B*(*x*_*k*_) ≥ 0 for all *k*, and (2*k* − 3 − 4*j*_*C*_) > 0 for *k* ≥ 2*j*_*C*_ + 2. Hence each term in the sum is non-negative, and the inequality is an equality if and only if *B* (*x*_*k*_) = 0 for all *k* ≥ 2*j*_*C*_ + 2. It therefore suffices to consider teams with competitive abilities contained in the range [0, ((2*j*_*C*_ + 1)/*M*)], subject to the constraint MCA ≤ *C* = (*j*_*C*_/*M*) + (1/2*M*). By the translation invariance of the problem as demonstrated in lemma 3.2, this is equivalent to the case in which $C=\frac{1}{2}$, and *M* is odd. ▪

**Proposition 3.16.**

*Assume that a collection of strategies* (*A*_1_, …, *A*_*n*_) *for the discrete game is an equilibrium point. Then they satisfy*

$$E(A_{k};A_{j})=0\;\forall j,k,\;E(A_{k};B)\geq 0\;\mathrm{for}\;\mathrm{any}\;\mathrm{strategy}\;B.$$

*Equivalently, each of* *A*_*k*_ *is an equilibrium strategy for the two-player game*.

**Proof.**

The proof is obtained from the proof for the bounded measurable and continuous games by an identical argument, by substituting *A*_*k*_ for *f*_*k*_ and *B* for *g*. ▪

## 4. Discussion

Games involving competing teams are widely researched and applied in numerous contexts; see [68,69] and references therein. Many authors have modelled competing teams as single players in the game theoretic sense [70–73]. Quoting [74], the ‘use of a two-person game to model conflict between groups presupposes that all group members have identical preferences over the set of possible outcomes and therefore that each group can be treated as a unitary player’. In biology, it has also been common to analyse competition between species by viewing the species as the player in the game theoretic sense [75]. Those approaches provide no mechanism to interpolate between the possibly diverse individuals of a team and the repercussions of the internal composition of the team for its competition with other teams.

Team games differ significantly from the player-to-player games that prevailed in the early days of game theory [76,77]. Our game, being non-cooperative, differs from cooperative game theory. According to [78], cooperative game theory models ‘the combination of specialized expertise within the team’. However, in the aforementioned work and many other studies based on cooperative game theory, teams do not necessarily compete with other teams [78–80]. Conflicting teams of cooperating players were also studied in [81] using graphs to describe connections between the teams. In contrast to our model, each team is valued by a ‘utility function’ on coalitions. Our teams may have different and dynamical sizes, which is a major difference to [79], which defined a team game as ‘a cooperative game in TU-form, whose values on coalitions of every cardinality but one are zero’. Many authors also investigate teams with non-cooperative game theory. However, there too, it is common to form teams without any actual competition between them. Examples include a selection process for team formations within a single sports club [82], in governance [83] or technology investment [84]. In [85], they propose a hybrid approach that bears some resemblance to ours. Their teams are collections of individuals with cost functions that depend on the actions of all players—including those of other teams. Considering a pair of teams, they ‘stipulate that the relationship between the two teams is completely adversarial and that cooperation between them is not permissible. In other words, both cooperation within each team and competition between the teams must coexist’. Conflicting teams of cooperating players were also studied in [81]. They define optimal solutions as states with Nash equilibria *between* teams such that each team’s strategy is Pareto optimal *within* the team itself. In contrast to this study, our teams need not have Pareto optimal strategies. As noted in the review [70], there is a general lack of multi-player games in conflict descriptions as most authors model conflicting agents as single players or assume that a conflict is a two-player multi-stage interaction. We believe that this motivates our study, as our game allows for each team a possibly large number of members.

Teams are important in evolutionary game theory, in which a standard approach is non-cooperative game theory [86,87]. Although teams refer to a constellation of individuals, the individuals composing a team in our model are an abstract concept. The individuals and team can represent any situation which satisfies the rules of our game. The motivation for non-cooperative game theory is that the individuals composing a team act independently. In numerous contexts, this is a reasonable assumption. For example, when the individuals in a team represent people, animals, microbes or other organisms, most spontaneous decisions are made without consulting others. If one considers the game as an aggregate over numerous decisions and subsequent consequences, then the majority of the actions taken by an individual are taken without consulting others. Although this may not be perfectly accurate, a similar assumption is made in modern portfolio theory [65,66], by assuming that the prices of distinct investment products are independent. This is not quite correct; it is a simplification that allows one to draw conclusions using the law of large numbers. The prices of investment products can be and often are correlated. Nonetheless, in spite of this imperfect simplification, modern portfolio theory remains widely in use today, indicating the utility of the theory, even if it is not perfect. Portfolio optimization uses game theory, both cooperative [21–23] and non-cooperative [24–28]. The constraint in our model on the mean competitive ability is similar to a budget constraint for the total value of a portfolio [29].

Our first contribution in this work is a game theoretic model that interpolates between the internal composition of a team and the repercussions for the team as a whole in competition with other teams. This model could be further developed and adapted to specific scenarios. It could also be combined with other competition models for teams in which the individuals are identical, like a plug-in which allows for different and diverse individuals and interpolates between the individual-level interactions and the team-level consequences. The second main contribution is the identification of all Nash equilibrium points and strategies. These strategies correspond to the most heterogeneous team composition. This indicates that a diverse team is a strong team in the face of competition with other teams. Our model is one dimensional in the sense that competitors are assigned scalar-valued competitive abilities, and all competitors are in the same competition. In other words, we do not consider competitions that involve several, parallel ‘abilities’. Such generalizations would need to invoke non-trivial dependence *between abilities*, because otherwise each ability, being independent, would adhere to the rules of our one-dimensional game. So, in fact, one could also apply our results to the case of multiple independent abilities and offer the same conclusion: the best strategy would be maximally diverse in each ability. We leave the question of several, cross-dependent abilities to future investigations and acknowledge that more research is required in order to understand such situations. It is important to note that in our model, the individuals in the teams are randomly paired to compete, implying a certain unpredictability. However, in other situations, in the face of one particular, predictable challenge, there may be an optimal strategy, known as an evolutionary stable strategy (ess) [88,89]. In such a situation, a homogeneous team comprising individuals characterized by an ess may be the strongest. This is not in contradiction, because one can show that in our model, there is *no* ess [45,63].

## 5. Conclusion

Seeking a theoretical explanation for the strength of diversity within a team, one could argue that a team is a type of biological system, whether the team represents a collection of people, animals, organisms or investment products. According to [90], ‘there exists in evolution a spontaneous tendency toward increased diversity and complexity, one that acts whether natural selection is present or not’. Those authors dubbed this the *Zero-Force Evolutionary Law*, or more colloquially, *Biology’s First Law*. Our results give a mechanistic underpinning for the strength of diversity that is broadly applicable due to its foundation in theoretical mathematics and that is consistent with the predictions of the fundamentals laws of biology and physics.

## Data Availability

This article has no additional data.
